# A novel numerical method for simulating a droplet in a Leidenfrost state on soft substrates

**DOI:** 10.1140/epje/s10189-026-00606-7

**Published:** 2026-07-27

**Authors:** Chen Miao, Florian Kummer

**Affiliations:** Mechanical Engineering, TU Darmstadt, Otto-Berndt-Str. 2, 64287 Darmstadt, Hessen Germany

## Abstract

**Abstract:**

This work presents a numerical framework for three-phase systems (liquid, gas, visco-elastic solid) using an extended discontinuous Galerkin (XDG) and level-set method. It simulates complex fluid–structure interactions with heat and mass transfer, maintaining strictly sharp phase interfaces without artificial smoothing. After verifying optimal convergence via a fluid–solid Taylor–Couette benchmark, the framework is applied to the Leidenfrost effect. While existing studies typically assume rigid solid surfaces, we investigate deformations induced by localized vapor pressure on soft substrates. We validate quasi-static droplet shapes against existing rigid solid models and derive a novel analytical extension for soft substrates using a Winkler foundation model. Our simulations demonstrate excellent agreement with this new analytical solution. Finally, the framework’s robustness is demonstrated by simulating a Leidenfrost droplet sliding down an inclined soft substrate, successfully capturing asymmetric droplet shape, substrate deformation and localized interfacial waves.

**Graphical abstract:**

**Figure:**
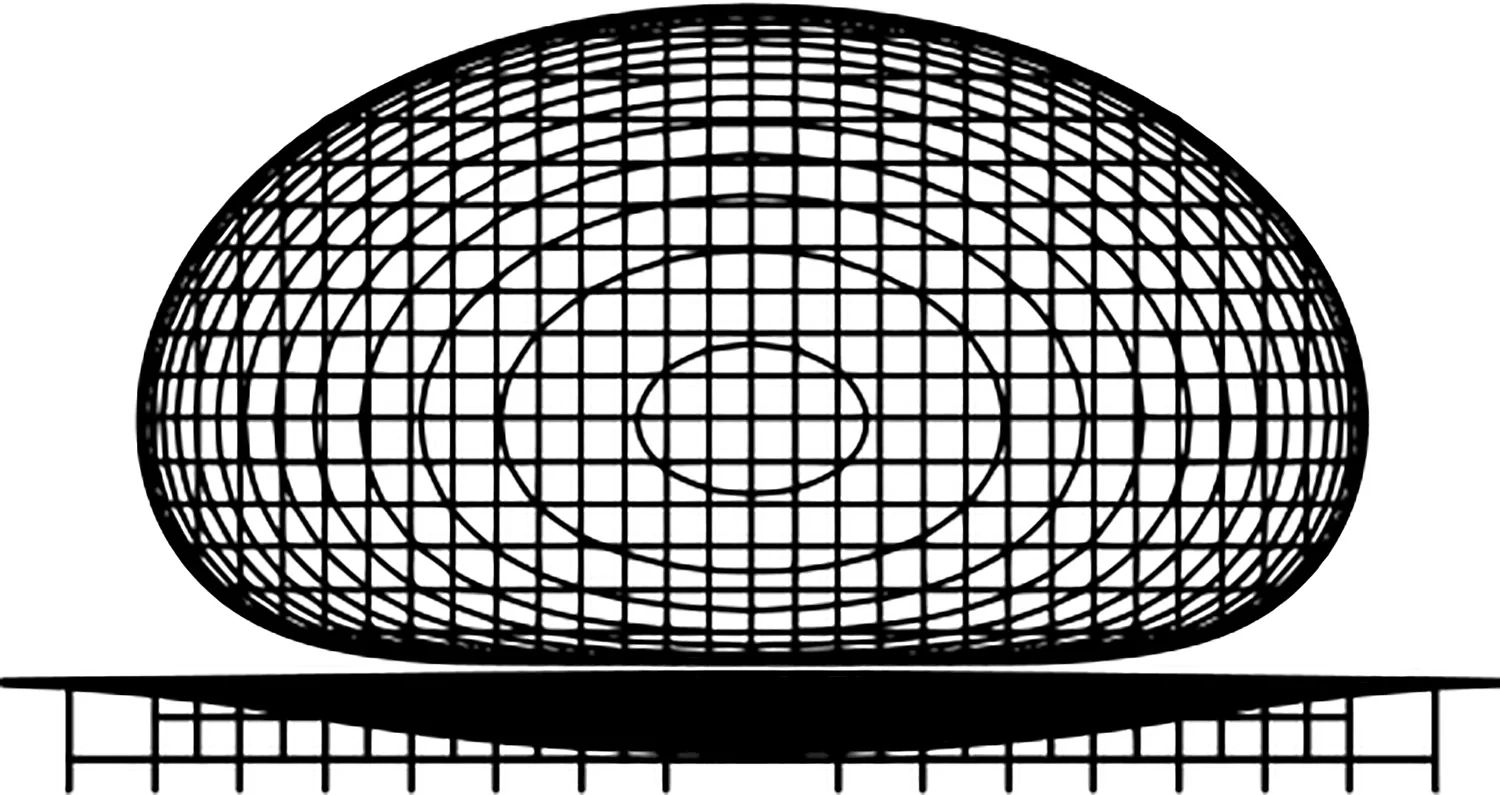

## Introduction

This work develops a numerical method for three-phase systems (liquid, gas and visco-elastic solid) featuring heat transfer and interfacial evaporation. We employ the extended discontinuous Galerkin (XDG) method to ensure a strictly *sharp* representation of phase interfaces and material properties, avoiding artificial smoothing. The method’s implementation and optimal convergence rates [1] are verified using a Taylor–Couette-like fluid–structure interaction benchmark

The validated framework is applied to the Leidenfrost effect [2]. Early studies established fundamental scaling behaviors [3] and vapor layer topologies [4], leading to accurate quasi-static analytical models [5–7].

Numerically, resolving both droplet-scale motion and the microscopic vapor film is notoriously expensive [8, 9]. Unlike coupled Navier–Stokes/lubrication approximations [10], our approach models both fluid phases with the full Navier–Stokes equations, coupled with simultaneous solid deformation and heat transfer. XDG’s high-order spatial discretization enables accurate thin-film resolution on comparatively less restrictive meshes.

While Leidenfrost substrates are typically assumed rigid and isothermal, elastocapillarity studies demonstrate that soft substrates fundamentally alter interfacial geometries and mechanical balances [11, 12]. These interactions are highly relevant to the field of dynamic wetting on adaptive, flexible and switchable substrates. Specifically, seminal finite-element methods for soft wetting have been introduced to numerically couple fluid dynamics with solid deformations, notably by the groups of van Brummelen [13], Aland [14] and Peschka [15]. This suggests localized high vapor pressure could deform a soft substrate, coupling fluid–solid interaction with heat transfer—a regime lacking systematic study.

Motivated by this gap, we investigate 3D Leidenfrost droplets on rigid and soft substrates. After validating the rigid quasi-static shape against analytical models [5], we propose an extension for soft substrates using a Winkler foundation model, an approach successfully used for sessile drops on elastic substrates by Snoeijer and co-workers [16]. By assuming solid displacement is proportional to applied pressure, we compare this analytical extension with our comprehensive 3D simulations before simulating a Leidenfrost droplet sliding down an inclined soft substrate.

## Mathematical model

For modeling evaporation of a droplet, which can either be separate or in contact with a soft or solid substrate, we consider the three-phase system consisting of the liquid droplet and the gas surrounding the droplet, both modeled as incompressible fluids, and the viscoelastic solid substrate, modeled as a Kelvin–Voigt material.

This is mathematically modeled as follows: The domain $\Omega \subset \mathbb {R}^D$, D=2 or D=3 is partitioned into three time-dependent subdomains $\Omega _\text {L}(t)$, $\Omega _\text {G}(t)$ and $\Omega _\text {S}(t)$, representing the liquid, gas and solid phase, respectively. These domains are separated by the liquid–gas interface $\mathcal {I}_{\text {L}\text {G}}(t)$ and the fluid–solid interface $\mathcal {I}_{F\text {S}}(t)$.

The interfaces are numerically represented as the zero-level set of scalar fields $\varphi _1(t,\boldsymbol{x})$ and $\varphi _2(t,\boldsymbol{x})$, for the sake of simplicity, also referred to level sets, i.e.,:

1a $$\begin{aligned} \mathcal {I}_{\text {L}\text {G}}(t)&= \{\boldsymbol{x} : \varphi _1(t,\boldsymbol{x}) = 0 \text {, } \varphi _2(t,\boldsymbol{x}) < 0 \} , \end{aligned}$$

1b $$\begin{aligned} \mathcal {I}_{F\text {S}}(t)&= \{\boldsymbol{x} : \varphi _2(t,\boldsymbol{x}) = 0 \} . \end{aligned}$$

The phase partition of the domain is then given by the relations:

2a $$\begin{aligned} \Omega _\text {L}(t)&= \{\boldsymbol{x} : \varphi _1(t,\boldsymbol{x})< 0 \text {, } \varphi _2(t,\boldsymbol{x}) < 0 \} , \end{aligned}$$

2b $$\begin{aligned} \Omega _\text {G}(t)&= \{\boldsymbol{x} : \varphi _1(t,\boldsymbol{x}) > 0 \text {, } \varphi _2(t,\boldsymbol{x}) < 0 \} , \end{aligned}$$

2c $$\begin{aligned} \Omega _\text {S}(t)&= \{\boldsymbol{x} : \varphi _2(t,\boldsymbol{x}) > 0 \} . \end{aligned}$$

In the fluid phases $\Omega _F(t)$, for $F\in \{\text {L}, \text {G}\}$, the incompressible Navier–Stokes equation applies,

3 $$\begin{aligned} \rho \left( \partial _t{\boldsymbol{u}} + \boldsymbol{u}\cdot \nabla {\boldsymbol{u}}\right) = -\nabla {p} + \mu \nabla \cdot { \left( \nabla {\boldsymbol{u}} + \nabla {\boldsymbol{u}}^{\text {T}}\right) } + \rho \boldsymbol{g} \end{aligned}$$

where $\boldsymbol{u}$ is the velocity field, *p* is the pressure and $\boldsymbol{g}$ is the gravity force. The solid phase $\Omega _\text {S}(t)$ is modeled as a viscoelastic material, in an Eulerian frame of reference, i.e.,

4a $$\begin{aligned} \rho \left( \partial _t{\boldsymbol{u}} + \boldsymbol{u}\cdot \nabla {\boldsymbol{u}}\right)&= -\nabla p + \mu \nabla \cdot (\nabla \boldsymbol{u}+ \nabla \boldsymbol{u}^T) \nonumber \\&\quad + \lambda \nabla \cdot ( \nabla \boldsymbol{r}+ \nabla \boldsymbol{r}^T ), \end{aligned}$$

4b $$\begin{aligned} \partial _t \boldsymbol{r}+ \textbf{u} \cdot \nabla \boldsymbol{r}&= \textbf{u}, \end{aligned}$$

where $\boldsymbol{r}$ denotes the displacement field. The evolution of temperature is governed by the usual heat equation defined over the entire domain Ω,

5 $$\begin{aligned} \rho c\left( \partial _t{T} + \boldsymbol{u}\cdot \nabla {T} \right) = k\nabla \cdot {\nabla {T}}, \end{aligned}$$

where T is the temperature field. The material parameters for the density ρ, viscosity μ, shear modulus λ, heat capacity c and thermal conductivity k are assumed to be constant within each subdomain.

Obviously, for both Eqs. (3) and (4), the incompressibility condition is required to close the system over the entire domain Ω, i.e.,

6 $$\begin{aligned} \nabla \cdot {\boldsymbol{u}} = 0 . \end{aligned}$$

The models in domains $\Omega _\text {L}(t)$, $\Omega _\text {G}(t)$ and $\Omega _\text {S}(t)$ are coupled through the interface conditions. The modeling of evaporation at the liquid–gas interface $\mathcal {I}_{\text {L}\text {G}}$ is employed in the respective jump conditions for the temperature and velocity fields.

7a $$\begin{aligned} \left[ \!\left[ {{\rho (\boldsymbol{u}- \boldsymbol{u}_{\mathcal {I}_{\text {L}\text {G}}})\cdot \textbf{n}_{\mathcal {I}_{\text {L}\text {G}}}}}\right] \!\right]&= 0 \end{aligned}$$

7b $$\begin{aligned} \left[ \!\left[ {{-p\boldsymbol{\textbf{I}} + \mu (\nabla {\boldsymbol{u}} + \nabla {\boldsymbol{u}}^{\text {T}})}}\right] \!\right]&\cdot \textbf{n}_{\mathcal {I}_{\text {L}\text {G}}}\nonumber \\ -\dot{m}\left[ \!\left[ {{\boldsymbol{u}}}\right] \!\right]&= -\sigma \kappa \textbf{n}_{\mathcal {I}_{\text {L}\text {G}}} \end{aligned}$$

7c $$\begin{aligned} T= T_s, \left[ \!\left[ {{T}}\right] \!\right]&= 0 \end{aligned}$$

7d $$\begin{aligned} \left[ \!\left[ {{ -k\nabla T}}\right] \!\right] \cdot \textbf{n}_{\mathcal {I}_{\text {L}\text {G}}}&= \dot{m}h_{vap} \end{aligned}$$

Here, $\boldsymbol{u}_{\mathcal {I}_{\text {L}\text {G}}}$ is the velocity of the interface, $\dot{m}$ is the mass flux due to evaporation, σ is the surface tension, κ is the curvature of the interface, $T_s$ is the saturation temperature and $h_{vap}$ is the latent heat of vaporization. The normal vector $\textbf{n}_{\mathcal {I}_{\text {L}\text {G}}}$ is given as $\textbf{n}_{\mathcal {I}_{\text {L}\text {G}}}= \frac{\nabla {\varphi _1}}{|\nabla {\varphi _1}|}$ and the jump operator $\left[ \!\left[ {{ - }}\right] \!\right]$ at any oriented interface $\mathcal {I}$ is defined as $\left[ \!\left[ {{f}}\right] \!\right] := f^+ - f^-$, where $f^+$ and $f^-$ denote the inside and outside value of *f*, with respect to the normal vector $\boldsymbol{n}_{\mathcal {I}}$, which defines the orientation of the interface.

At the interfaces between fluid and solid phases $\mathcal {I}_{F\text {S}}$, the continuity of velocity, stress, temperature and heat flux is imposed:

8a $$\begin{aligned} \llbracket \boldsymbol{u}\rrbracket&= 0, \end{aligned}$$

8b $$\begin{aligned} \llbracket - p \textbf{I} + \mu ( \nabla \boldsymbol{u}+ \nabla \boldsymbol{u}^{\textrm{T}} ) \qquad \qquad  &   \nonumber \\ + \lambda ( \nabla \boldsymbol{r}+ \nabla \boldsymbol{r}^{\textrm{T}} ) \rrbracket \cdot \textbf{n}_{\mathcal {I}_{F\text {S}}}&= 0, \end{aligned}$$

8c $$\begin{aligned} \llbracket T\rrbracket&= 0, \end{aligned}$$

8d $$\begin{aligned} \left[ \!\left[ {{ -k\nabla T}}\right] \!\right] \cdot \textbf{n}_{\mathcal {I}_{F\text {S}}}&= 0, \end{aligned}$$

where λ, the shear modulus (Lamé’s second parameter), appears in the stress jump operator due to the viscoelastic properties of the solid phase. For the fluid phases, λ=0. Note that the normal vector $\textbf{n}_{\mathcal {I}_{F\text {S}}}= \frac{\nabla {\varphi _2}}{|\nabla {\varphi _2}|}$ is defined to point from the fluid phase toward the solid phase.

With respect to the setups presented in Sect. 4, we partition the boundary into a top, a side and a bottom region, i.e., $\partial \Omega = \partial \Omega _t \cup \partial \Omega _s \cup \partial \Omega _{b}$, corresponding to the locations in the computational domain. To close the system (3)–(6), we employ zero-stress, free-slip and no-slip conditions and for the heat equation (5), we impose Dirichlet, Neumann and Dirichlet conditions on top, side and bottom, respectively. In detail:

$$\begin{aligned} (-p\textbf{I} + \mu \left( \nabla {\boldsymbol{u}} + \nabla {\boldsymbol{u}}^{\text {T}}\right) ) \cdot \textbf{n}_{\partial \Omega }&= 0&\text {on } \partial \Omega _t, \\ T&= T_{\text {sat}}&\text {on } \partial \Omega _t, \\ \boldsymbol{u}\cdot \textbf{n}_{\partial \Omega }&= 0&\text {on } \partial \Omega _s, \\ \textbf{P}_{\partial \Omega } \left( \nabla {\boldsymbol{u}} + \nabla {\boldsymbol{u}}^{\text {T}}\right) \cdot \textbf{n}_{\partial \Omega }&= 0&\text {on } \partial \Omega _{s,\text {G}}, \\ \textbf{P}_{\partial \Omega } \left[ \mu (\nabla {\boldsymbol{u}} + \nabla {\boldsymbol{u}}^{\text {T}}) + \right.&\\ \left. \lambda (\nabla {\boldsymbol{r}} + \nabla {\boldsymbol{r}}^{\text {T}}) \right] \cdot \textbf{n}_{\partial \Omega }&= 0&\text {on } \partial \Omega _{s,\text {S}}, \\ \boldsymbol{u}&= 0&\text {on } \partial \Omega _b, \\ T&= T_{\text {wall}}&\text {on } \partial \Omega _b, \end{aligned}$$

where $\textbf{P}_{\partial \Omega } = \textbf{I} - \textbf{n}_{\partial \Omega } \otimes \textbf{n}_{\partial \Omega }$ denotes the tangential projection operator and $\partial \Omega _{s,\text {G}} = \partial \Omega _s \cap \Omega _\text {G}(t)$ and $\partial \Omega _{s,\text {S}} = \partial \Omega _s \cap \Omega _\text {S}(t)$ denote the side boundaries of the gas and solid phase, respectively.

## Numerical method

The two main pillars of the numerical method are, first, a level-set method for tracking the position of the interface, c.f. Sect. 3.2, and second, an extended discontinuous Galerkin (XDG) method for the spatial discretization, c.f. Sect. 3.3. At first, for the sake of completeness, the defining properties of an XDG method are briefly summarized.

### Extended discontinuous Galerkin approximation

We consider a triangulation of the computational domain Ω into *background* cells $K_j$, $j = 1, \ldots , N_{\text {cells}}$, which do not overlap and cover the entire domain Ω. For each phase $s\in \{\text {L}, \text {G}, \text {S}\}$, we define the cut-cell as the intersection of the cell with the corresponding domain:

9 $$\begin{aligned} K_{j,s}(t) := K_j \cap \Omega _s(t) . \end{aligned}$$

These cut-cells represent the “active region” of each background cell for the respective phase.

***Cell agglomeration***

Since the interfaces can cut the background cells in an arbitrary way, cut-cells can be arbitrarily small, which can lead to instabilities of the numerical method if no special care is taken. One major stabilization technique is Ghost-Penalty stabilization, introduced by Burman [17], which adds additional consistent stabilization terms to the variational formulation. Another stabilization technique, which is used in this work, is cell agglomeration, which means that small cut-cells, i.e., if their volume fraction $\frac{|K_{j,s}(t)|}{|K_j|}$ is below a certain threshold α, are merged with neighboring cells to form larger agglomerated cells. Cell agglomeration can be efficiently implemented for XDG methods in three dimensions [18]. One advantage of cell agglomeration is that it does not require any modification of the variational formulation. Since the agglomerated XDG space is a subspace of the “original” XDG space, the variational formulation is still consistent with the original PDEs, and no additional stabilization terms are required. Due to the sub-space structure, the agglomeration procedure can also be implemented in a non-intrusive way, using projection and prolongation operators similar to those which can be found in a multigrid method.

***Agglomerated XDG space***

Based on the agglomerated cut-cells $K_{j,s}^{\alpha }(t)$, for an agglomeration threshold α, we define the time-dependent extended discontinuous Galerkin space of polynomial degree *k* as:

10 $$\begin{aligned} \mathbb {P}_{k}^{\text {X},\alpha }(t) := \left\{ v(\boldsymbol{x}) : \, v(\boldsymbol{x})|_{K_{j,s}^{\alpha }(t)} \in \mathcal {P}_k \, \forall j, s\right\} , \end{aligned}$$

where $\mathcal {P}_k$ denotes the space of multivariate polynomials of degree at most *k*. Thus, functions in $\mathbb {P}_{k}^{\text {X},\alpha }(t)$ are allowed to be discontinuous across phase boundaries and across mesh cell interfaces, while maintaining polynomial smoothness within each cut-cell.

For the coupled three-phase system, search for a numerical approximation of the solution in the product space of the respective XDG spaces for displacement, velocity, pressure and temperature fields. I.e., one searches for tuples $\boldsymbol{U}= (\boldsymbol{r}, \boldsymbol{u}, p, T)$ in the product space

11 $$\begin{aligned} \mathbb {V}_{k}^{\text {X},\alpha }(t)&:= (\mathbb {P}_{k}^{\text {X},\alpha }(t))^D \times (\mathbb {P}_{k}^{\text {X},\alpha }(t))^D \nonumber \\&\quad \times \mathbb {P}_{k-1}^{\text {X},\alpha }(t) \times \mathbb {P}_{k}^{\text {X},\alpha }(t) . \end{aligned}$$

Note that the pressure field is approximated in a lower-order space, which is a common choice to ensure the inf-sup stability of the velocity–pressure pair.

***XDG basis functions***

A canonical way to construct a XDG basis is to start with a standard DG basis on the background mesh and then restrict it to the cut-cells: Let $\phi _{j,n}(\boldsymbol{x})$ be a “standard” DG basis function on the background mesh. Here, *j* corresponds to the cell $K_j$ and *n* to the local basis function index within the cell. Basis functions for the respective cut-cells $K_{j,\text {L}}(t)$, $K_{j,\text {G}}(t)$, and $K_{j,\text {S}}(t)$ are then obtained by restricting the standard DG basis functions to these cut-cells, e.g., as

$$\begin{aligned} \phi _{j,\text {L},n} (t,\boldsymbol{x}):=&\phi _{j,n}(\boldsymbol{x})&H(-\varphi _1(t, \boldsymbol{x})) H(-\varphi _2(t, \boldsymbol{x})), \\ \phi _{j,\text {G},n} (t,\boldsymbol{x}):=&\phi _{j,n}(\boldsymbol{x})&H(+\varphi _1(t, \boldsymbol{x})) H(-\varphi _2(t, \boldsymbol{x})), \\ \phi _{j,\text {S},n} (t,\boldsymbol{x}):=&\phi _{j,n}(\boldsymbol{x})&H(+\varphi _2(t, \boldsymbol{x})), \end{aligned}$$

where H(-) denotes the Heaviside function.

***Numerical integration in cut-cells***

With respect to the implementation, it is important to understand that this restriction, i.e., the multiplication of the standard DG basis functions with the Heaviside functions, is not performed explicitly in the implementation. The restriction of a basis polynomial to a certain cut-cell is instead, implicitly, achieved by the numerical integration rules, which constructed—on the fly, in each time step—only integrate over the respective cut-cell. In cells which are cut by both interfaces, we employ a method proposed by Beck and Kummer [19]. In cells which are cut by only one interface, Saye’s method [20, 21] is used.

***A basis for the product space***

Finally, a basis of the product space $\mathbb {V}_{k}^{\text {X},\alpha }(t)$, to represent tuples $\boldsymbol{U}$, is obtained in the canonical way by combining these scalar basis functions with the unit vectors $\boldsymbol{e}_d \in \mathbb {R}^{2 D + 2}$. I.e., let $1 \le \beta \le 2 D + 2$ be a component index, where the first *D* components correspond to the displacement field, the next *D* components correspond to the velocity field, followed by pressure and temperature components, respectively. Then, a vector basis function can be defined as

12 $$\begin{aligned} \boldsymbol{\Phi }_{j,\beta ,s,n}(t,\boldsymbol{x}) := \boldsymbol{e}_\beta \phi _{j,s,n}(t,\boldsymbol{x}) . \end{aligned}$$

Using this basis, one can represent the solution tuple in terms of this basis, i.e.,

13 $$\begin{aligned} \boldsymbol{U}(t,\boldsymbol{x}) = \sum _{j, \beta , s, n} \tilde{U}_{j,\beta ,s,n}(t) \boldsymbol{\Phi }_{j,\beta ,s,n}(t,\boldsymbol{x}) . \end{aligned}$$

Note that in this notation the range of mode index *n* actually depends on the component β, and the phase s, since the polynomial degree of the pressure is one lower than the other fields. Furthermore, the phase index s also depends on the cell index *j*, since not all cells are cut by all interfaces, and thus not all phases are present in each cell.

### Level-set method and velocity extension

The interfaces of the three-phase system are tracked using the level-set method. The evolution of the level sets $\varphi _1(t, \boldsymbol{x})$ (for the liquid–gas interface) and $\varphi _2(t, \boldsymbol{x})$ (for the fluid–solid interface) is modeled by the advection equation defined over the entire computational domain Ω:

14 $$\begin{aligned} \partial _t{\varphi _i} + \nabla \cdot { ( \varphi _i \cdot \boldsymbol{u}_{\text {ex},i} ) } = 0, \end{aligned}$$

where $\boldsymbol{u}_{\text {ex},i}$ denotes the velocity field for the advection of the respective interface.

It should be noted that the setting $\boldsymbol{u}_{\text {ex},i} = \boldsymbol{u}$ is not a viable choice, for the following reasons:

- For i=1, i.e., for the liquid–gas interface $\mathcal {I}_{\text {L}\text {G}}(t)$, given by $\varphi _1$, the velocity $\boldsymbol{u}$ has a jump in normal direction at the interface, due to the presence of phase change, and the interface velocity $\boldsymbol{u}_{\mathcal {I}_{\text {L}\text {G}}}$ is only given at the interface itself.
- For i=2, i.e., for the fluid–solid interface $\mathcal {I}_{F\text {S}}(t)$, given by $\varphi _2$, the velocity $\boldsymbol{u}$ typically has a pronounced kink at the interface, i.e., a jump in its gradient, due to the discontinuity in material properties and governing equations. This is incompatible with the requirement of a smooth level-set function, which requires a regular velocity field for the advection of the level set.

Note that the latter argument can also be expressed in a more formal way: The level-set function $\varphi _i$ is typically discretized in a standard discontinuous Galerkin space $\mathbb {P}_{k}$, which is defined in analog fashion to the XDG space in Eq. (10), but without the cut-cell construction, i.e., on the background mesh. Using a velocity field $\boldsymbol{u}$ from an XDG space, i.e., with a jump or a kink at the interface, would result in a low accuracy of the level-set advection, due to the incompatibility of the function spaces for $\varphi _i$ and $\boldsymbol{u}$.

A divergence-free extension velocity field can actually be obtained as the solution of a steady-state Stokes problem, with an interior Dirichlet boundary condition at the interface:

15a $$\begin{aligned} - \Delta \boldsymbol{u}_{\text {ex},i} + \nabla {\psi }&= 0, \end{aligned}$$

15b $$\begin{aligned} \nabla \cdot {\boldsymbol{u}_{\text {ex},i}}&= 0, \end{aligned}$$

15c $$\begin{aligned} \boldsymbol{u}_{\text {ex},i}&= s_{\mathcal {I},i} \boldsymbol{n}_i \text { at } \mathcal {I}_i \end{aligned}$$

15d $$\begin{aligned} \left( -\psi \textbf{I} + \nabla \boldsymbol{u}_{\text {ex},i} \right) \cdot \boldsymbol{n}_{\partial \Omega }&= \boldsymbol{0} \quad \text {at } \partial \Omega \end{aligned}$$

For the liquid–gas interface, i.e., i=1, $\mathcal {I}_1 = \mathcal {I}_{\text {L}\text {G}}$, $\vec {\textit{n}}_1 = \textbf{n}_{\mathcal {I}_{\text {L}\text {G}}}$ the interface-normal velocity is $s_{\mathcal {I},i} = \boldsymbol{u}_{\mathcal {I}} \cdot \textbf{n}_{\mathcal {I}_{\text {L}\text {G}}}$, where $\boldsymbol{u}_{\mathcal {I}_{\text {L}\text {G}}}$ is obtained from the jump condition in Eq. (7a). For the fluid–solid interface, i.e., i=2, $\mathcal {I}_2 = \mathcal {I}_{F\text {S}}$, $\vec {\textit{n}}_2 = \textbf{n}_{\mathcal {I}_{F\text {S}}}$, the interface-normal velocity is $s_{\mathcal {I},2} = \boldsymbol{u}\cdot \textbf{n}_{\mathcal {I}_{F\text {S}}}$, since $\boldsymbol{u}$ is continuous at the fluid–solid interface. This Stokes problem is solved in a standard DG space $\mathbb {P}_{k}^D \times \mathbb {P}_{k-1}$. The interior Dirichlet boundary condition (15c) is enforced by an additional penalty term $\oint _{ \mathcal {I}_i } \eta ( \boldsymbol{u}_{\text {ex},i} - s_{\mathcal {I},i} ) \cdot \boldsymbol{m} \ \textrm{d} S$, where $\boldsymbol{m}$ is the test function for the momentum and η is a penalty parameter, as is common in symmetric interior penalty methods [22, 23], see also Appendix A. For the numerical implementation of the surface integral, the same quadrature rules as for the XDG method are used, see Sect. 3.1

The main advantage of this Stokes extension approach is that the conservative form of the level-set equation (14) can be used, which has been found superior to the non-conservative form in terms of long-time stability. However, regarding configurations with evaporation, one should note that the divergence-free constraint (15b) is inconsistent with the interface velocity condition (15c) if the interface $\mathcal {I}_{\text {L}\text {G}}$ is closed and the mass flux $\dot{m}$ is nonzero. After discretization, this leads to an over-determined system. However, at least for the comparatively low evaporation rates considered in this work, see also Sect. 4, we found that the discrete system can be computed with the direct sparse linear solver PARDISO [24], which provides a least-squares solution in the case of an over-determined system, therefore distributing the error across the entire domain. Spot checks of the resulting velocity field give confidence that the interface velocity condition (15c) is fulfilled with sufficient accuracy.

An alternative approach, which avoids these complications, would be to entirely remove the divergence-free constraint (15b), solve only a harmonic equation for the extension velocity and use a non-conservative form of (14). This, however, has shown other challenges regarding numerical stability, for which we have not found a satisfactory solution yet. We want to emphasize that the level-set method and the construction of extension velocities, in the context of the XDG method, especially for multi-phase problems with evaporation, are still an open research question and will be the issue of future works.

### Spatial discretization

Within this section, a time-continuous, but space-discrete formulation of the numerical method is presented. The XDG discretization of the governing equations, (3, 4, 5, 6) together (8) for the liquid–gas coupling and (8) for the fluid–solid coupling, is given as: For a specific point *t* in time, one searches a velocity–displacement–pressure–temperature tuple $(\boldsymbol{r}, \boldsymbol{u}, p, T) \in \mathbb {V}_{k}^{\text {X},\alpha }(t)$, which fulfills:

16 $$\begin{aligned}&\int _{\Omega }\partial _t \boldsymbol{r}\cdot \boldsymbol{o} + \rho ( \partial _t \boldsymbol{u}\cdot \boldsymbol{m} + c( \partial _t T) \tau ) \textrm{d} V\nonumber \\&\quad + B_h(\boldsymbol{r}; \boldsymbol{r}, \boldsymbol{u}, p, T; \boldsymbol{o}, \boldsymbol{m}, l, \tau ) = 0, \end{aligned}$$

for all test function tuples $(\boldsymbol{o},\boldsymbol{m},l,\tau ) \in \mathbb {V}_{k}^{\text {X},\alpha }(t)$.

The form $B_h(\ldots )$ is linear in the test functions as well as in the trial functions T and p, but nonlinear in $\boldsymbol{r}$ and $\boldsymbol{u}$. It can be split into a sum of terms corresponding to the different phases, as well as coupling terms, i.e.,

17 $$\begin{aligned} B_h(\boldsymbol{r}_0; \boldsymbol{r}, \boldsymbol{u},&p, T; \boldsymbol{o}, \boldsymbol{m}, l, \tau ) = \nonumber \\&\quad B_h^{\text {L},\text {G}}(\boldsymbol{u}, p, T; \boldsymbol{o}, \boldsymbol{m}, l, \tau ) \nonumber \\&\qquad + \int _{\Omega _F(t)} \boldsymbol{r}\cdot \boldsymbol{o} \ \textrm{d} V \nonumber \\&+ B_h^{\text {S}} (\boldsymbol{r}_0; \boldsymbol{r}, \boldsymbol{u}, p, T; \boldsymbol{o}, \boldsymbol{m}, l, \tau ) \nonumber \\&+ B_h^{\mathcal {I}_{F\text {S}}} (\boldsymbol{r}_0; \boldsymbol{r}, \boldsymbol{u}, p, T; \boldsymbol{o}, \boldsymbol{m}, l, \tau ). \end{aligned}$$

Note that in $B_h (\ldots )$, a splitting of the displacement field into a known part $\boldsymbol{r}_0$, used in the momentum equation, i.e., all terms with test function $\boldsymbol{m}$, and an unknown part $\boldsymbol{r}$, used in the displacement transport, i.e., the term with test function $\boldsymbol{o}$, is employed. This ad-hoc linearization has been found to be beneficial for the condition number of the resulting linear system and also for the convergence of the nonlinear solver.

The form $B_h^{\text {L},\text {G}}(\ldots )$ corresponds to the fluid phases and their respective coupling. It can be found in the work of Rieckmann et al. [25] and is omitted for brevity. The formulation can be traced back to the work of Kummer [26] and furthermore, to the textbook of Di Pietro and Ern [27]. Also, since Eq. (3) can be obtained from the solid model in Eq. (4) by removing all terms related to displacement, we only present the solid phase form $B_h^{\text {S}} (\ldots )$. The second term enforces the displacement $\boldsymbol{r}$ to be zero in the fluid phases.

The solid phase form can be decomposed further, i.e.,

18 $$\begin{aligned} B_h^{\text {S}} (\boldsymbol{r}_0; \boldsymbol{r},&\boldsymbol{u}, p, T; \boldsymbol{o}, \boldsymbol{m}, l, \tau ) = \nonumber \\&\quad c_h(\boldsymbol{u}, \boldsymbol{u}, \boldsymbol{m}) + b_h(p, \boldsymbol{m}) \nonumber \\&\qquad + a_h(\mu , \boldsymbol{u}, \boldsymbol{m}) + a_h(\lambda , \boldsymbol{r}_0, \boldsymbol{m}) \nonumber \\&+ c_h(\boldsymbol{r}, \boldsymbol{u}, \boldsymbol{o}) \nonumber \\&+ c_h(\boldsymbol{u}, cT, \tau ) + a_h(k, T, \tau ) \nonumber \\&- b_h(l, \boldsymbol{u}) \end{aligned}$$

Here, $a_h(\ldots )$, $b_h(\ldots )$ and $c_h(\ldots )$ are the standard bilinear forms for the diffusion, pressure–velocity coupling and convection terms, respectively. Their definition is given in Appendix A.

The coupling term $B_h^{\mathcal {I}_{F\text {S}}}(\ldots )$ weakly enforces the interface conditions from Eq. (8) on the fluid–solid interface $\mathcal {I}_{F\text {S}}(t)$. In particular, it accounts for continuity of velocity and temperature, as well as continuity of normal stress and heat flux across the interface. To this end, we employ a Nitsche-type formulation, in which consistency terms arise from integration by parts on both sides of the interface, adjoint-consistent terms provide symmetry, and penalty contributions stabilize the jumps of the primal variables. The exact definition is also given in Appendix A.

### Temporal discretization

The semi-discrete formulation in Eq. (16) can be converted into a system of coupled ordinary differential equations (ODEs) in time for the degrees-of-freedom $\tilde{U}_{j,\beta ,s,n}$ of the solution field $\boldsymbol{U}$, c.f. Eq. (12), by inserting the vector basis functions $\boldsymbol{\Phi }_{j,\beta ,s,n}$ into Eq. (16). Due to continuity condition (6), which contains no time derivative, it can be written as a differential-algebraic equation of the form

19 $$\begin{aligned} \mathcal {M} \partial _t \tilde{U} + \mathcal {N}(\boldsymbol{r}_0, \tilde{U}) = 0, \end{aligned}$$

with a singular mass matrix $\mathcal {M}$ which is zero in those rows and columns which correspond to the continuity equation, resp., the pressure degrees-of-freedom. The nonlinear operator $\mathcal {N}(\boldsymbol{r}_0, \tilde{U})$ is defined by the variational form $B_h(\ldots )$, i.e.,

$$\begin{aligned} \mathcal {N}(\boldsymbol{r}_0, \tilde{U}) := \left[ \begin{array}{c} \vdots \\ B_h(\boldsymbol{r}_0; \boldsymbol{U}(t,-); \boldsymbol{\Phi }_{i,\alpha ,s',m}) \\ \vdots \end{array} \right] _{ \forall i, \alpha , s' , m } . \end{aligned}$$

To integrate one time step from a known initial state at time $t^0$, consisting of the initial states for the level-set fields $\varphi ^0_1$ and $\varphi ^0_2$, as well as the initial state for the solution tuple $\boldsymbol{U}^0$, to a new state at time $t^1$, the following steps are performed:

1. At first, the extension velocities $\boldsymbol{u}_{\text {ex},1}$ and $\boldsymbol{u}_{\text {ex},2}$ are obtained, as described in Sect. 3.2.
2. An explicit time step of the level-set advection equation (14) is performed, using a standard Runge–Kutta method of order 3 to obtain the new level-set fields $\varphi _1$ and $\varphi _2$ at time $t^1$.
3. The new XDG space $\mathbb {V}_{k}^{\text {X},\alpha }(t^1)$ is constructed based on the new level-set fields $\varphi _1$ and $\varphi _2$. The initial state $\boldsymbol{U}^0$ is projected onto the new XDG space $\mathbb {V}_{k}^{\text {X},\alpha }(t^1)$. The ad-hoc linearization around $\boldsymbol{r}_0$ is extracted from this projected state.
4. A backward Euler time discretization of the ODE system (19) is performed, which results in a nonlinear system of equations for the unknown state $\boldsymbol{U}^1$. A nonlinear solver [28] is employed to solve the nonlinear system of equations (19) and obtain the new state $\boldsymbol{U}^1$ at time $t^1$.

## Results

This section evaluates the performance and accuracy of the proposed numerical method through a series of increasingly complex test cases. First, the fluid–solid Taylor–Couette flow with heat transfer (see Sect. 4.1) is employed to verify the numerical implementation against known analytical solutions. Subsequently, the model is benchmarked against established literature results for the Leidenfrost effect on a rigid substrate, as detailed in Sect. 4.2. In Sect. 4.3, we extend the investigation to soft substrates. The analytical prediction for the Leidenfrost droplet profile is introduced and serves as the basis for verifying our numerical results. Finally, the capability of the method to handle complex, dynamic interactions is demonstrated in Sect. 4.4, which simulates a Leidenfrost droplet sliding down a soft substrate under the influence of gravity.

### Fluid–solid Taylor–Couette flow with heat transfer

The Taylor–Couette flow describes the motion of a fluid between two rotating coaxial cylinders with an inner radius $r_i$ and an outer radius $r_a$. In this fluid–structure interaction (FSI) case involving heat transfer, a fluid–solid interface $\mathcal {I}_{F\text {S}}(t)$ is established at a radius $r_{\textit{FS}}$, where $r_i< r_{\textit{FS}} < r_a$.

When the Taylor number (*Ta*) and Rayleigh number (*Ra*) remain significantly below the critical threshold of 1707.76, both Taylor vortex formation and Rayleigh–Bénard convection are suppressed [29, 30]. Under these conditions, the fluid and solid dynamics are invariant along the axial direction. Consequently, the system is reduced to a 2D representation in the *xy*-plane.

The solid domain is defined as $\Omega _S = \{ \boldsymbol{x} \in \mathbb {R}^2 \mid r_i< \Vert \boldsymbol{x}\Vert _2 < r_{\textit{FS}} \}$, and the fluid domain is given by $\Omega _F = \{ \boldsymbol{x} \in \mathbb {R}^2 \mid r_{\textit{FS}}< \Vert \boldsymbol{x}\Vert _2 < r_a \}$. The boundary conditions for velocity and temperature at the inner and outer cylinders are specified as:

$$\begin{aligned}{\left\{ \begin{array}{ll} \textbf{u} \cdot \textbf{e}_{\theta } = u_i, \quad T = T_i &  \text {for } r = r_i, \\ \textbf{u} \cdot \textbf{e}_{\theta } = u_a, \quad T = T_a &  \text {for } r = r_a, \end{array}\right. } \end{aligned}$$

where $r = \sqrt{x^2 + y^2}$ and $\textbf{e}_{\theta } = (-y, x)^T / r$ is the tangential unit vector. At the fluid–solid interface $\mathcal {I}_{F\text {S}}(t)$ when $r=r_{FS}$, the coupling conditions derived in the theoretical section are enforced. These include the continuity of velocity equation (8a) and temperature equation (8c), as well as the continuity of stress equation (8b) and heat flux equation (8d).

The specific physical parameters employed for this benchmark are:

$$\begin{aligned}\begin{aligned} r_i&= 0.5,&r_a&= 2.0,&r_{\textit{FS}}&= (\sqrt{2}/2 + 2) / 2, \\ u_i&= 0.0,&u_a&= 1.0, \\T_i&= 1.0,&T_a&= 5.0, \\ \rho _{F}&= 10.0,&\rho _{S}&= 1.3, \\ \mu _{F}&= 0.2,&\mu _{S}&= 0.2, \\ \lambda _{F}&= 0.0,&\lambda _{S}&= 10.0, \\ k_{F}&= 3.0,&k_{S}&= 40.0. \end{aligned} \end{aligned}$$

The exact solutions for the velocity $\textbf{u}_{\text {ex}}$, pressure $p_{\text {ex}}$, displacement $\textbf{r}_{\text {ex}}$, and temperature $T_{\text {ex}}$ are expressed as functions of the radial coordinate *r*:

$$\begin{aligned}\begin{aligned} \textbf{u}_{\text {ex}}(\boldsymbol{x})&= u_{\theta }(r) \textbf{e}_{\theta }, \quad&\textbf{r}_{\text {ex}}(\boldsymbol{x})&= r_{\theta }(r) \textbf{e}_{\theta }, \\ p_{\text {ex}}(\boldsymbol{x})&= p(r), \quad&T_{\text {ex}}(\boldsymbol{x})&= T(r), \end{aligned} \end{aligned}$$

where the radial distributions of the velocity, displacement, pressure, and temperature fields are defined piecewise for the solid and fluid regions. The radial distributions are given by:

- Solid Region ($$r_i \le r \le r_{\textit{FS}}$$):
  $$\begin{aligned} \begin{aligned} u_{\theta }(r)&= 0, \\ r_{\theta }(r)&= 0.135217492469620 r \\&\quad -0.033804373117405 / r, \\ p(r)&= 0, \\ T(r)&= 0.645015726004179 \ln (r) \\&\quad + 1.447090831896623. \end{aligned} \end{aligned}$$
- Fluid Region ($$r_{\textit{FS}} \le r \le r_a$$):
  $$\begin{aligned} \begin{aligned} u_{\theta }(r)&= 0.922554663967561 r \\  &\quad -1.690218655870246 / r, \\ p(r)&= 0.553219620205395 r^2 \\  &\quad -4.054229670655003 \ln (r) \\&\quad - 1.856945418023684 / r^2 \\  &\quad + 1.227350228287268, \\ T(r)&= 8.600209680055716 \ln (r) \\  &\quad -0.961211091954969. \end{aligned} \end{aligned}$$

For the evaluation of the spatial convergence of the proposed method, a grid refinement study is performed on the computational domain $\Omega = (-2, 2)^2 \setminus (-0.5, 0.5)^2$. In contrast to all other test cases in this work, only Dirichlet boundary conditions for velocity and temperature are applied. The domain is discretized using a sequence of successively refined uniform Cartesian grids, ranging from 8×8 up to 256×256 cells. For each mesh resolution, transient simulations are performed until a steady state is achieved. The accuracy of the numerical framework is then assessed by computing the $L^2$ error norms between the numerical and exact analytical solutions.

**Fig. 1:**
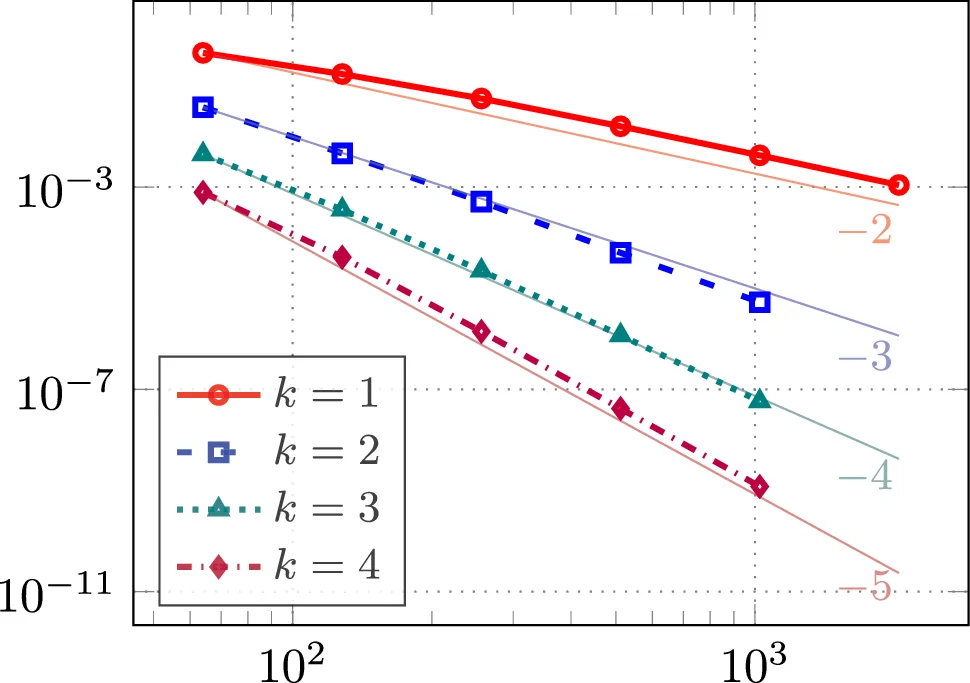
Temperature field $L^2$ error vs. number of cells for different DG polynomial degrees *k*

Figure 1 illustrates the convergence behavior of the temperature field, which exhibits a clear k+1 convergence order. The convergence rates for the velocity, pressure, and displacement fields are also computed and summarized in Table 1. The results indicate that the numerical errors decay in strong agreement with theoretical expectations, with the pressure field exhibiting a convergence rate one order lower than those of velocity and displacement, consistent with the use of a lower-degree basis polynomial for the pressure approximation. This benchmark serves as a critical verification of the spatial numerical discretization, demonstrating its accuracy and robustness.

**Table 1:** Calculated convergence rates for velocity $(u_x, u_y)$, pressure (*p*), displacement $(r_x, r_y)$, and temperature (*T*) using discontinuous Galerkin methods with basis polynomial degree *k*

*k*	$u_x$	$u_y$	*p*	$r_x$	$r_y$	*T*
1	1.68	1.68	1.02	1.64	1.64	1.75
2	3.15	3.15	2.32	3.03	3.03	3.21
3	4.09	4.09	3.25	4.18	4.18	4.07
4	5.06	5.06	3.60	4.82	4.82	4.86

### Leidenfrost on hard substrate

Previous studies on the quasi-static shape of Leidenfrost droplets have predominantly focused interacting with rigid substrates, where the substrate deformation and heat transfer within the substrate are neglected. In this section, we validate our numerical method against the analytical solution, which is derived in [5]. To ensure a direct comparison, the thermal conductivity of the substrate is set to a very large value, and the shear modulus of the substrate is set to a very large value, effectively modeling a rigid substrate with negligible temperature gradients.

**Fig. 2:**
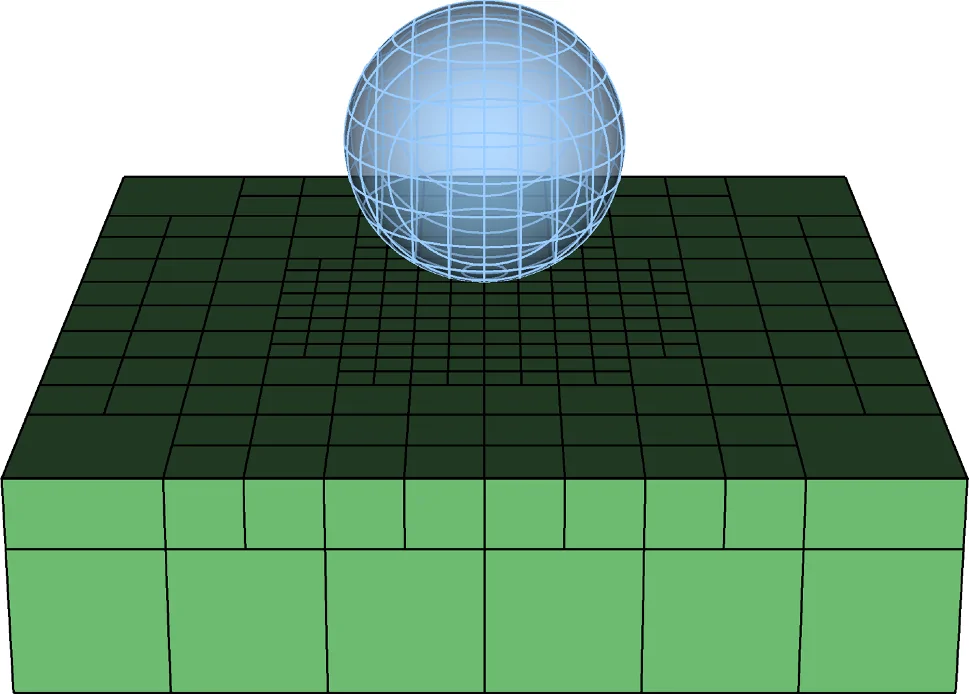
Initial configuration of the 3D simulation for a Leidenfrost droplet on a rigid substrate in Cartesian coordinates

As Fig. 2 illustrates the initial configuration of the simulation, it is performed in a 3D Cartesian coordinate system, with the substrate located at the bottom of the computational domain. The simulation is initialized, on the first time step, on a base grid of $6 \times 6 \times 6$ cells with 2 levels of Adaptive Mesh Refinement (AMR) around the liquid–gas interface, i.e., each original cell is split up into $8^2$ cells. In the first couple of time steps, this is progressively refined up to AMR level 4 and dynamically adapted if the interfaces move. The AMR level is allowed to differ only by one between neighboring cells, which results in transitional refinement layers. Under the influence of gravity, the initially spherical droplet falls toward the substrate. Evaporation prevents the droplet from making direct contact with the substrate, leading to the formation of a vapor layer that supports the droplet. It continues to evaporate and adjust its shape until a steady state is reached, at which point the shape of the droplet is analyzed and compared to the analytical solution. The time step size is defined by the capillary time step criterion [25]. Boundary conditions are set as described at the end of Sect. 2, with $T_{\text {wall}} = 5$ and $T_{\text {sat}} = 0$.

**Fig. 3:**
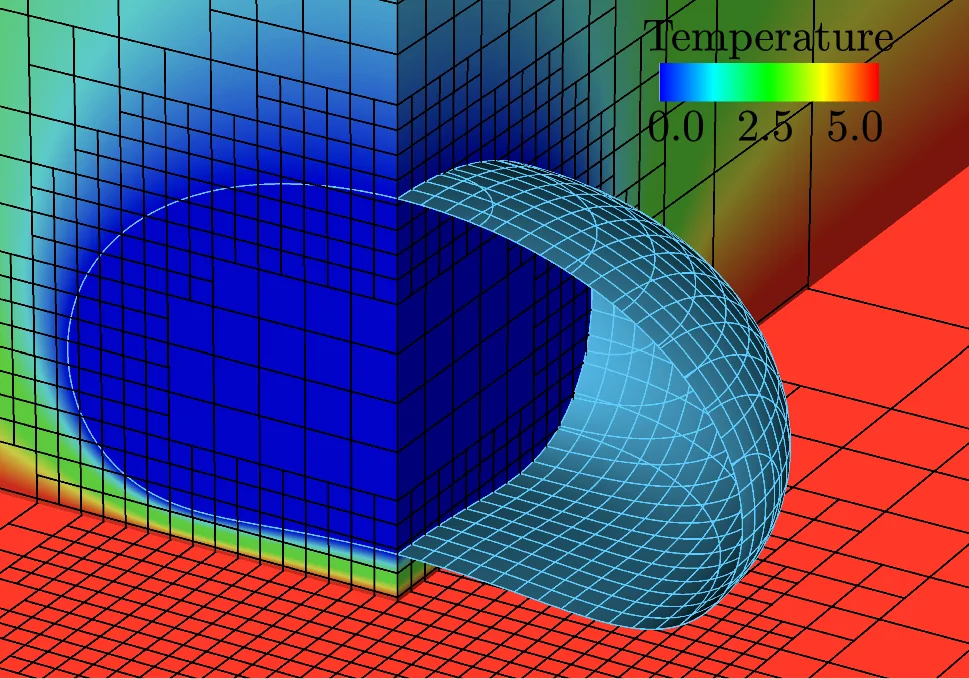
Cross-sectional view of the droplet shape and temperature field after reaching steady state, for $r^*_{max}/l_c^* = 1.7$

After reaching the steady state, the maximum radius of the droplet is defined as $r^*_{max}$, which can be non-dimensionalized by the capillary length scale $l_c^*= \sqrt{\sigma ^* / (\rho _L^* g^*)}$. Apart from the maximum radius, the shape of the droplet is also influenced by the evaporation number $\mathcal {E}$, which is defined in Eq. (29). The evaporation number $\mathcal {E}$ is set to be 0.0007 for simulations in this section.

Figure 3 shows a cross-sectional view of the droplet shape together with the temperature field at (quasi-) steady state, where the volume loss is negligible within the simulated timeframe, similar to other works [5–7, 10]. Since the thermal conductivity of the substrate is set to a very large value, the temperature in the substrate is nearly uniform and remains the temperature at the bottom domain boundary.

**Fig. 4:**
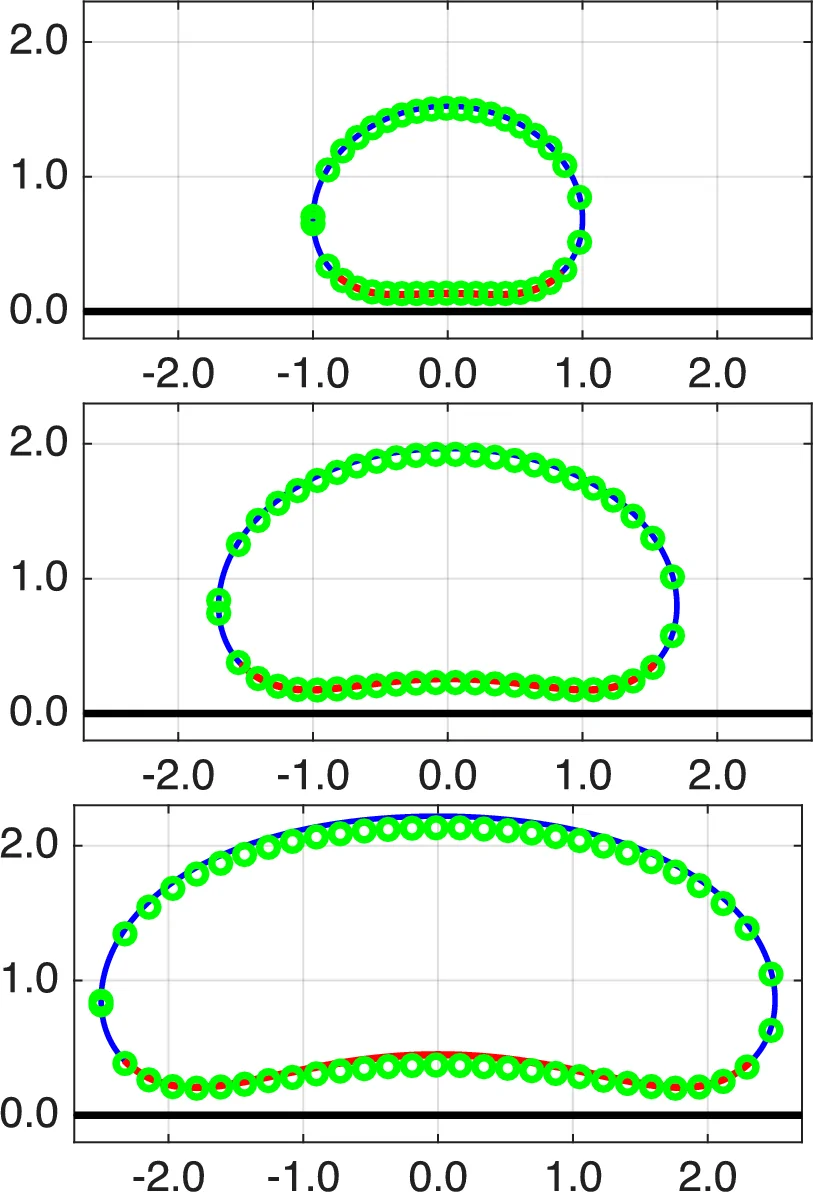
Comparison of the shape of the Leidenfrost droplet between analytical solution from [5] (lines) and simulation (dots). From top to bottom: $r^*_{max} / l_c^* = 1.0$, 1.7 and 2.5

The droplet shapes with different sizes are compared with the analytical solution for three cases. As shown in Fig. 4, the numerical results, indicated by green dots, are in excellent agreement with the analytical solution, represented by the blue and red lines for the upper and lower interfaces, respectively. This agreement confirms the accuracy of our numerical method in capturing the shape of Leidenfrost droplets on rigid substrates.

### Leidenfrost on soft substrate

To the best of our knowledge, the exact shape of a static Leidenfrost droplet on a soft substrate has not yet been described in the literature. To investigate this problem, we derive an analytical description of the droplet shape and use it to validate our numerical method.

#### Analytical solution

**Fig. 5:**
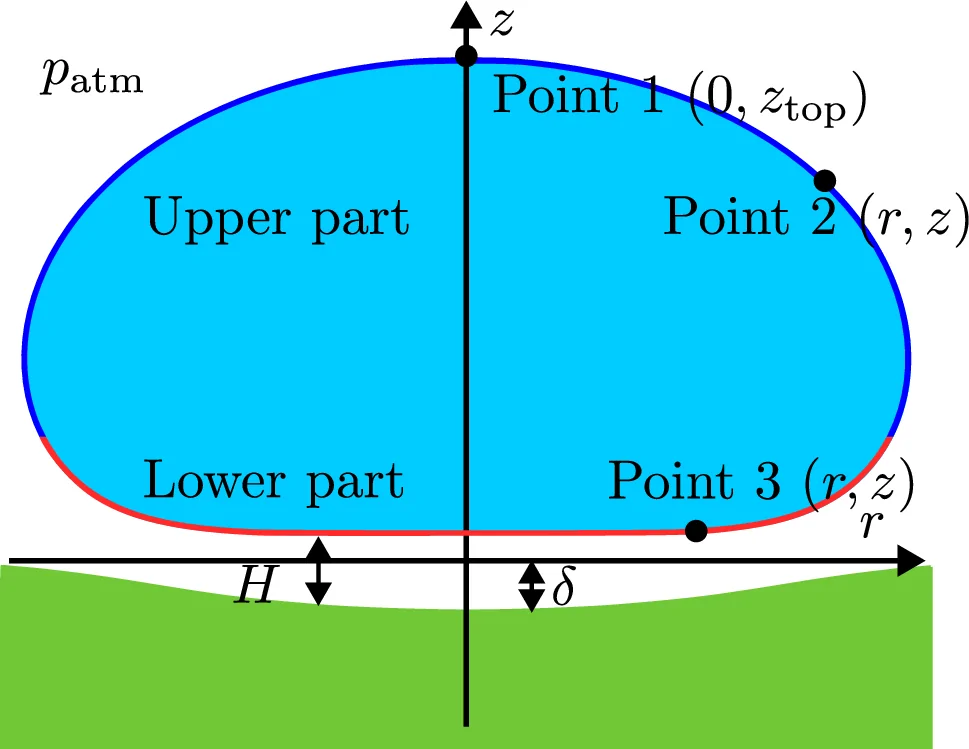
Cross-sectional view of the axisymmetric droplet profile. Points 1, 2 are located in the upper part of the droplet, while Point 3 is located in the lower part

Following the approach in [5, 6], the droplet is assumed to remain at the saturation temperature, and internal liquid motion is neglected. As shown in Fig. 5, the droplet profile is divided into upper and lower regions and is assumed to be axisymmetric about the *z*-axis.

In the upper part of the droplet, gas motion is also neglected, so the gas pressure is assumed to be constant and equal to $p_{\text {atm}}$. The droplet shape is therefore governed by the balance between hydrostatic and capillary pressures. Accordingly, the pressure $p^*$ at Point 2 can be written as:

20 $$\begin{aligned} \begin{aligned} p^*(r^*, z^*)&= p_{\text {atm}}^* + \sigma ^* \kappa ^* \\&= p_{\text {atm}}^* + \sigma ^* \kappa _{\text {top}}^* + \rho _L^* g^* (z_{\text {top}}^* - z^*). \end{aligned} \end{aligned}$$

Non-dimensionalization is performed using the capillary length scale $l_c^*= \sqrt{\sigma ^* / (\rho _L^* g^*)}$. Dropping the asterisk for dimensionless quantities, we define:

21 $$\begin{aligned} r = \frac{r^*}{l_c^*}, \quad z = \frac{z^*}{l_c^*}, \quad \kappa = \kappa ^* \cdot l_c^*, \end{aligned}$$

by utilizing this definition, Eq. (20) simplifies directly to:

22 $$\begin{aligned} \kappa _{\text {top}} + z_{\text {top}} = \kappa (r) + z(r). \end{aligned}$$

Here, κ represents the local curvature and *z* denotes the vertical height. Accordingly, a dimensionless pressure variable π is defined as:

23 $$\begin{aligned} \pi (r) = \kappa (r) + z(r), \end{aligned}$$

which remains constant throughout the upper part of the droplet: $\pi _{\text {top}} = \pi (r)$.

For the lower part, the liquid droplet is still assumed to be motionless and at constant saturation temperature. For the soft solid phase, the Winkler model is used for the substrate below the Leidenfrost droplet, which assumes that the small displacement δ of the substrate surface is proportional to the vapor pressure at the fluid–solid interface. After non-dimensionalization, the pressure at Point 3 in the gas phase can be expressed as:

24 $$\begin{aligned} p_v=p_{\text{ atm } }+ \kappa _{\text{ top } }+\left( z_{\text{ top } }-z\right) - \kappa . \end{aligned}$$

The vertical displacement of the substrate surface, caused by the vapor pressure at the fluid–solid interface, can be calculated by the Winkler model as: $\delta ^* = p^*_v / k_s^*$, where $k_s^*$ is the Winkler modulus, can be calculated by Young’s modulus [31]. After non-dimensionalization, the displacement can be expressed as:

25 $$\begin{aligned} \delta \left( r\right) =\frac{\rho _L^*g^*}{k_s^*}\left( p_v-p_{a t m}\right) =\alpha (\pi _{top}-\pi (r)), \end{aligned}$$

in which $\alpha = \rho _L^* g^* / k_s^*$ is a dimensionless elasto-gravity number, which represents the ratio of gravitational force to elastic force. A larger value of α indicates a softer substrate that deforms more under the droplet, while α=0 corresponds to a perfectly hard (rigid) substrate.

Thus, the real thickness of the vapor layer beneath the droplet is given by:

26 $$\begin{aligned} H(r)=z(r)+\delta (r)=z(r)+\alpha (\pi _{\text{ top } }-\pi (r)). \end{aligned}$$

Inside the vapor layer, based on conservation of mass, the vapor outflow driven by the pressure gradient must be supplied by the evaporative flux driven by heat conduction, which can be expressed as:

27 $$\begin{aligned} \frac{1}{r^*} \frac{\partial }{\partial r^*}\left( r^* \rho _v^*\frac{-1}{12} \frac{H^{* 3} d p_v^*}{\mu ^*_{v} d r^*}\right) =\frac{k_v^* \Delta T^*}{\mathcal {L}^* H^*}. \end{aligned}$$

Using Eq. (24) for pressure gradient, the resulting governing equation for the vapor layer after non-dimensionalization is:

28 $$\begin{aligned} \frac{1}{12} \frac{1}{r} \frac{\partial }{\partial r}\left( r H^3 \frac{\partial \pi }{\partial r}\right) -\frac{\mathcal {E}}{H}=0 \end{aligned}$$

where $\mathcal {E}$ is the evaporation number,

29 $$\begin{aligned} \mathcal {E}=\frac{k_v^* \mu _v^* \Delta T^*}{\sigma ^* \rho _v^* \ell _c^* \mathcal {L}^*}, \end{aligned}$$

the curvature κ can be calculated by:

30 $$\begin{aligned} \kappa =\frac{\frac{\partial ^2 z}{\partial r^2}+\frac{1}{r}\left[ 1+\left( \frac{\partial z}{\partial r}\right) ^2\right] \frac{\partial z}{\partial r}}{\left[ 1+\left( \frac{\partial z}{\partial r}\right) ^2\right] ^{3 / 2}}. \end{aligned}$$

As a result, to calculate the static analytical shape of the Leidenfrost droplet and the deformation of the soft substrate under the assumptions described above, only three parameters should be provided: the maximum radius $r_{max}$, evaporation number $\mathcal {E}$ and the elasto-gravity number α. For α=0, which means H(r)=z(r), the equation reduces to the case in reference [5, 10] for a rigid substrate.

Similarly, the analytical shape of a Leidenfrost droplet on a fluid substrate can also be calculated. The shape of the fluid substrate itself can be determined by balancing hydrostatic and capillary pressures. However, the detailed analysis is beyond the scope of this paper.

#### Numerical solution

**Fig. 6:**
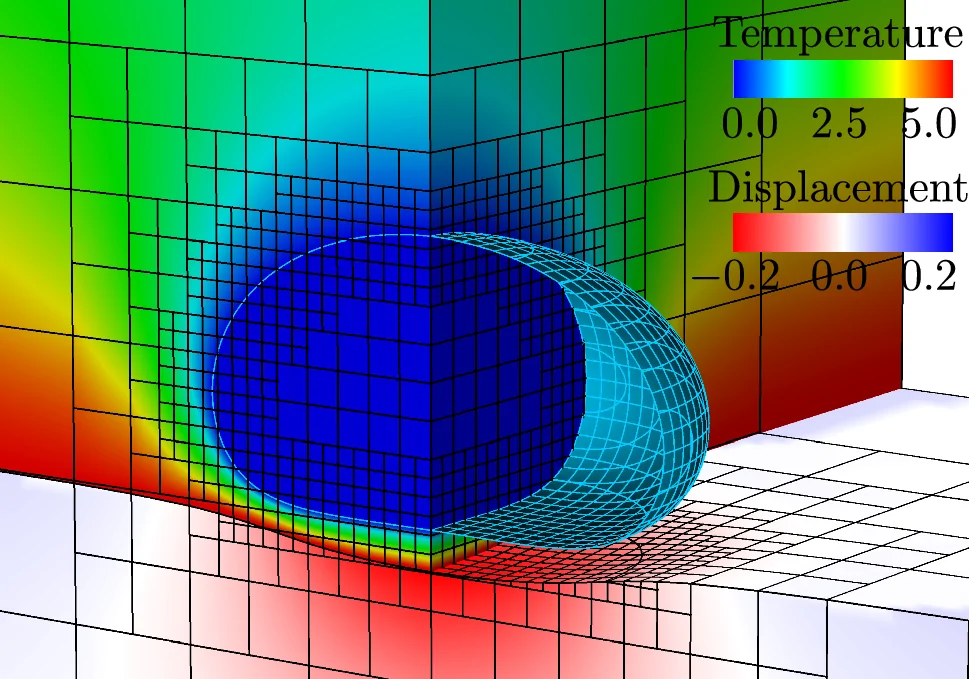
Cross-sectional view of the droplet shape, substrate deformation and temperature field after reaching steady state, for $r_{max} = 1.7$

For numerical simulation, the equations mentioned in Sect. 2 are solved without the assumptions for the analytical solution in Sect. 4.3.1. The settings for grid and time step sizes, boundary conditions are same as in Sect. 4.2. In Fig. 6, the droplet shape and substrate deformation at steady state are shown for a case with $r_{max}=1.7$, $\mathcal {E}=0.0007$, and α=0.09565. The droplet shape is similar to the case on a rigid substrate, but with a slightly flattened bottom. The substrate deformation directly below the droplet is significant.

**Fig. 7:**
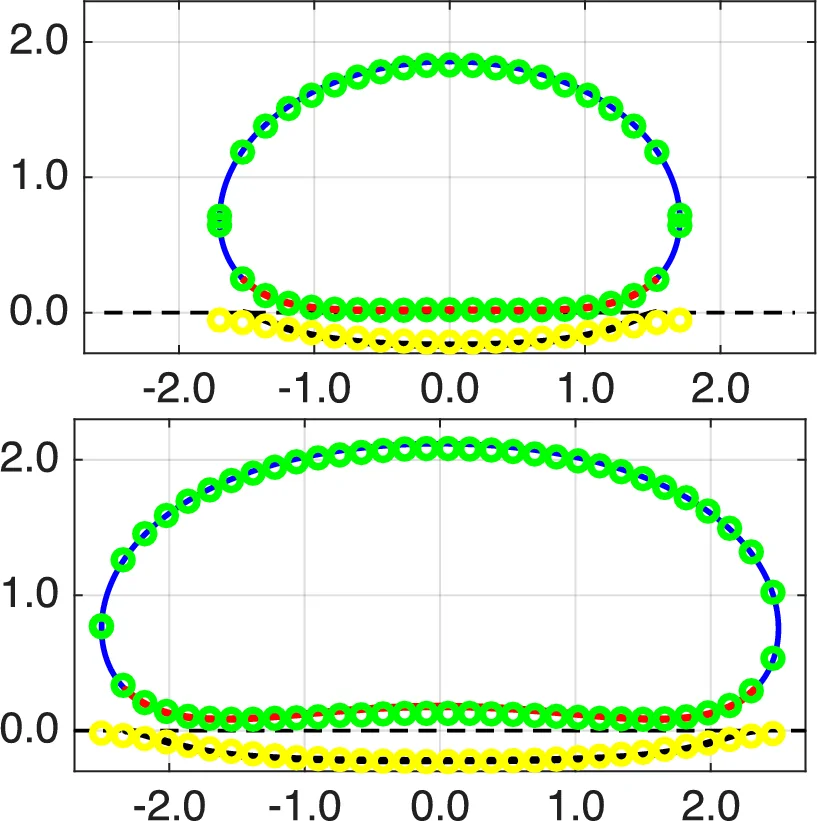
Comparison of the shape of the Leidenfrost droplet and substrate deformation between analytical solution (lines) and simulation (dots). From top to bottom: $r_{max} = 1.7$ and 2.5

A comparison between the analytical and simulated shapes is presented in Fig. 7, where the analytical solution is represented by blue and red lines for the upper and lower droplet interfaces, respectively, and the substrate deformation is represented by the solid black line, and the initial undeformed substrate surface is represented by the thin dashed black line. The numerical results are indicated by green dots for the droplet shape and yellow dots for the substrate deformation.

For the substrate area near or outside the droplet’s edge, the limitations of the Winkler model become apparent. The assumption of independent local deformation neglects the shear coupling inherent in continuous elastic media, thereby failing to capture the smooth non-local deformation. However, in the region directly beneath the droplet, the Winkler model provides an accurate approximation.

Overall, the numerical results show excellent agreement with the analytical solution for both the droplet shape and substrate deformation, particularly in the region directly beneath the droplet where the deformation is most significant.

### Leidenfrost sliding

**Fig. 8:**
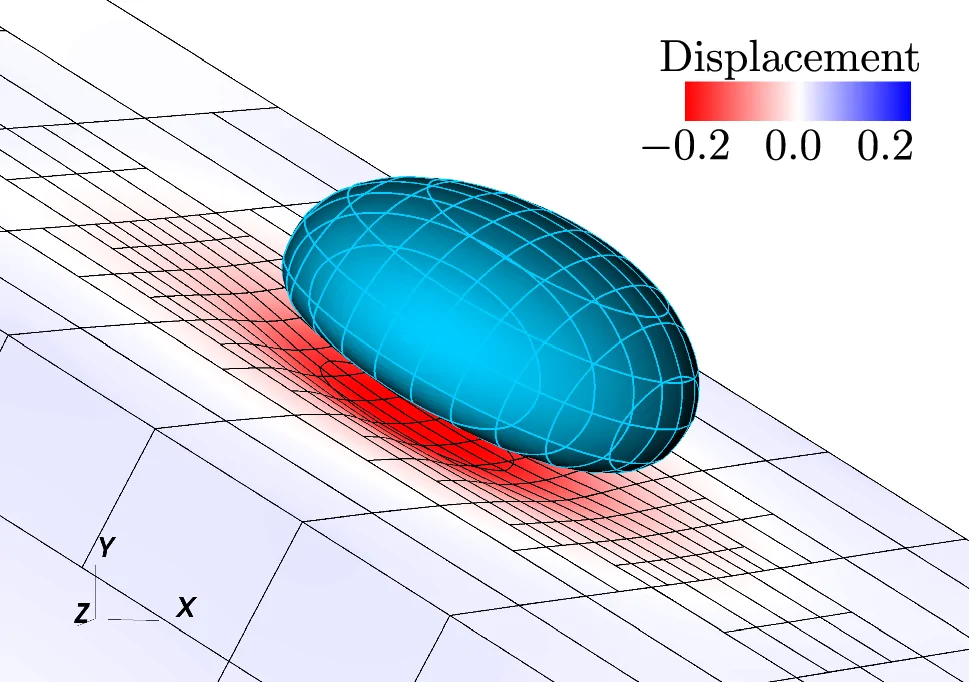
Zoomed-in view of the droplet shape and substrate deformation during sliding

In the final test case, we investigate the dynamics of a Leidenfrost droplet impacting and subsequently sliding down a soft substrate inclined at 30 degrees. The simulation is performed with similar parameters as in Sec. 4.3.2. The spatial domain is discretized using a base grid of $2 \times 2 \times 12$ cells combined with AMR level of 4 around the liquid–gas interface. Figure 8 shows a snapshot at the instant when the droplet rebounds and begins to slide. The droplet shape and substrate deformation are similar to the static case in Fig. 6, but during sliding the droplet develops a larger radius at the front edge and a smaller radius at the rear edge.

During the sliding process, waves continue to form at the fluid–solid interface behind the droplet and propagate upward along the substrate surface. We suspect that these are Rayleigh waves induced by the sliding droplet and the vapor pressure acting on the substrate, although further analysis is required to confirm this hypothesis.

## Conclusion

In this work, we developed a numerical framework for three-phase systems consisting of two fluid phases and one solid phase. By combining a level-set approach with an extended discontinuous Galerkin (XDG) discretization, the method enables a strictly sharp representation of both liquid–gas and fluid–solid interfaces.

The framework’s optimal convergence for velocity, pressure, displacement, and temperature fields is first verified using a fluid–solid Taylor–Couette benchmark. We then simulate the static shape of Leidenfrost droplets. For rigid substrates, our results agree well with the analytical solution in [5]. For soft substrates, we derived a new analytical solution based on the Winkler model, which matches our numerical results excellently, particularly for the region directly beneath the droplet. Finally, we simulated a Leidenfrost droplet sliding down an inclined soft substrate under gravity, successfully capturing the droplet’s asymmetric deformation and the induced interfacial waves.

Future work could improve the analytical model by incorporating non-local elastic deformations for soft substrates. We also plan to investigate Leidenfrost droplets bouncing on soft substrates, where sustained bouncing might occur [32]. Further analysis is needed to characterize the sliding-induced interfacial waves and confirm whether they are indeed Rayleigh waves. Furthermore, combining this framework with soft wetting will enable the simulation of more complex physical processes on adaptive, flexible and switchable substrates.

## Data Availability

The code used for the simulations in this work is available at Zenodo [33].

## Variational forms for the spatial discretization

This section contains the definition of the variational forms for the spatial discretization, used e.g., in Eq. (18). We present the forms in a minimal way, for sake of completeness. For a more comprehensive presentation we refer to other works [25–27]. We also restrict ourselves to the forms for the solid phase. In the fluid phase, similar or equivalent forms are used, which can also be found in the afore-mentioned references. The following notation is used: Let $\Gamma _s$ be the skeleton of the mesh, i.e., the union of all faces of the elements in the solid domain $\Omega _\text {S}$. The vector $\boldsymbol{n}$ denotes a normal vector on these faces, pointing in an arbitrary but fixed direction. As for the interfaces $\mathcal {I}_{\text {L}\text {G}}$ and $\mathcal {I}_{F\text {S}}$, the jump operator $\left[ \!\left[ {{-}}\right] \!\right]$ denotes the difference of some property between the inner and the outer value, w.r.t. the normal vector $\boldsymbol{n}$. Furthermore, $\left\{ {-}\right\}$ denotes the average of these values.

The viscous form, for vector fields and a diffusion coefficient μ, is given as:

A1 $$\begin{aligned} a_h (\mu , \boldsymbol{v}, \boldsymbol{m})&= \int _{\Omega _\text {S}} \mu ( \nabla \boldsymbol{v} : \nabla \boldsymbol{m} + \left( \nabla \boldsymbol{v}\right) ^T : \nabla \boldsymbol{m} )\, \textrm{d} V \nonumber \\ - \oint _{\Gamma _\text {S}\setminus \partial \Omega _{s}}&\mu \left\{ {\nabla \boldsymbol{v} + \nabla \boldsymbol{v}^T }\right\} \boldsymbol{n} \cdot \left[ \!\left[ {{ \boldsymbol{m} }}\right] \!\right] \, \textrm{d} S \nonumber \\ - \oint _{\Gamma _\text {S}\setminus \partial \Omega _{s}}&\mu \left\{ {\nabla \boldsymbol{m} + \nabla \boldsymbol{m}^T }\right\} \boldsymbol{n} \cdot \left[ \!\left[ {{ \boldsymbol{v} }}\right] \!\right] \, \textrm{d} S \nonumber \\ + \oint _{\Gamma _\text {S}\setminus \partial \Omega _{s}}&\eta \mu \left[ \!\left[ {{ \boldsymbol{v} }}\right] \!\right] \cdot \left[ \!\left[ {{ \boldsymbol{m} }}\right] \!\right] \, \textrm{d} S \nonumber \\ - \oint _{ \partial \Omega _{s,\text {S}}}&\mu (\boldsymbol{n} \cdot (\nabla \boldsymbol{v} + \nabla \boldsymbol{v}^T ) \boldsymbol{n} ) \cdot ( \boldsymbol{m} \cdot \boldsymbol{n} ) \, \textrm{d} S \nonumber \\ - \oint _{ \partial \Omega _{s,\text {S}}}&\mu (\boldsymbol{n} \cdot (\nabla \boldsymbol{m} + \nabla \boldsymbol{m}^T ) \boldsymbol{n} ) \cdot ( \boldsymbol{v} \cdot \boldsymbol{n} ) \, \textrm{d} S \nonumber \\ + \oint _{\partial \Omega _{s,\text {S}}}&\eta \mu ( \boldsymbol{v} \cdot \boldsymbol{n} ) \cdot ( \boldsymbol{m} \cdot \boldsymbol{n}) \, \textrm{d} S \end{aligned}$$

This is a standard symmetric interior penalty (SIP) formulation, going back to the work of Arnold [22, 23], which is modified to discretize symmetric tensors $\nabla \boldsymbol{v} + (\nabla \boldsymbol{v})^T$. The last three lines correspond to the free-slip boundary condition on the side walls [34]. The penalty parameter η depends on the polynomial degree of the basis functions and the inverse of a cell length scale. Specifically, it is computed, in each agglomerated cell $K_{j,s}^{\alpha }$, as $\tilde{\eta } = \eta _0 k^2 \frac{ | \partial K_{j,s}^{\alpha } | }{ | K_{j,s}^{\alpha } | },$ with a base penalty factor $\eta _0 = 4$. On each face of the mesh, the penalty parameter is then defined as the maximum of the values of $\tilde{\eta }$ in the adjacent cells [26].

The pressure–velocity coupling, as well as ensuring that the velocity is divergence-free, is realized via the form $b_h(\ldots )$:

A2 $$\begin{aligned} b_h(p, \textbf{m})= -\int _{\Omega _\text {S}} p \nabla \cdot \textbf{m} \textrm{d} V +\oint _{\Gamma _\text {S}} \left\{ { p }\right\} \textbf{n} \cdot \left[ \!\left[ {{ \textbf{m} }}\right] \!\right] \textrm{d} S . \end{aligned}$$

The convection form for a vector property $\boldsymbol{w}$ is given as:

A3 $$\begin{aligned} c_h(\boldsymbol{w}, \boldsymbol{v}, \boldsymbol{m}) =&- \int _{\Omega _s}(\boldsymbol{w} \otimes \boldsymbol{v}): \nabla _h \boldsymbol{m} \textrm{d} V \nonumber \\&+ \oint _{\Gamma _\text {S}} \textbf{L}(\boldsymbol{w}, \boldsymbol{v}) \cdot \left[ \!\left[ {{ \boldsymbol{m} }}\right] \!\right] \textrm{d} S \end{aligned}$$

with upwind flux $\textbf{L}$:

A4 $$\begin{aligned} \textbf{L}(\boldsymbol{w}, \boldsymbol{v}) = {\left\{ \begin{array}{ll} \left( \boldsymbol{w}^{-} \otimes \{\boldsymbol{v}\}\right) \boldsymbol{n} &  \text{ if } \{\boldsymbol{v}\} \cdot \boldsymbol{n} \ge 0 \\ \left( \boldsymbol{w}^{+} \otimes \{\boldsymbol{v}\}\right) \boldsymbol{n} &  \text{ otherwise } \end{array}\right. } , \end{aligned}$$

where the superscripts $\boldsymbol{w}^{-}$ and $\boldsymbol{w}^{+}$ denote the values of $\boldsymbol{w}$ on the inside, resp. outside of the face, w.r.t. the normal vector $\boldsymbol{n}$. The scalar convection form, as well as the scalar diffusion form, can also be defined in a similar way, by replacing the vector properties in Eq. (A1) and Eq. (A3) with scalar properties.

Finally, the coupling form $B_h^{\mathcal {I}_{F\text {S}}} (\ldots )$ is given as:

A5 $$\begin{aligned} B_h^{\mathcal {I}_{F\text {S}}}&(\boldsymbol{r}_0; \boldsymbol{r}, \boldsymbol{u}, p, T; \boldsymbol{o}, \boldsymbol{m}, l, \tau ) = \nonumber \\ \oint _{\mathcal {I}_{F\text {S}}(t)}&\textbf{L}(\boldsymbol{u}, \boldsymbol{u}) \cdot \left[ \!\left[ {{ \boldsymbol{m} }}\right] \!\right] \nonumber \\&+\left\{ { p \textbf{I} }\right\} \textbf{n}_{\mathcal {I}_{F\text {S}}}\cdot \left[ \!\left[ {{\boldsymbol{m}}}\right] \!\right] \nonumber \\&-\left\{ { \mu ( \nabla \boldsymbol{u}+ \nabla \boldsymbol{u}^T )}\right\} \textbf{n}_{\mathcal {I}_{F\text {S}}}\cdot \left[ \!\left[ {{\boldsymbol{m}}}\right] \!\right] \nonumber \\&-\left\{ { \mu ( \nabla \boldsymbol{m} + \nabla \boldsymbol{m}^T )}\right\} \textbf{n}_{\mathcal {I}_{F\text {S}}}\cdot \left[ \!\left[ {{\boldsymbol{u}}}\right] \!\right] \nonumber \\&+ \eta \max (\mu ^{-}, \mu ^{+}) \left[ \!\left[ {{ \boldsymbol{u}}}\right] \!\right] \cdot \left[ \!\left[ {{ \boldsymbol{m} }}\right] \!\right] \nonumber \\&-( \lambda ( \nabla \boldsymbol{r}+ \nabla \boldsymbol{r}_0^T ))^{+} \textbf{n}_{\mathcal {I}_{F\text {S}}}\cdot \boldsymbol{m}^{+} \nonumber \\&-( \lambda ( \nabla \boldsymbol{m} + \nabla \boldsymbol{m}^T ))^{+} \textbf{n}_{\mathcal {I}_{F\text {S}}}\cdot \boldsymbol{r}_0^{+} \nonumber \\&+( \boldsymbol{r}\otimes \boldsymbol{u})^{+} \cdot \boldsymbol{o}^{+} \nonumber \\&+ \textbf{L}(T, \boldsymbol{u}) \cdot \left[ \!\left[ {{ \tau }}\right] \!\right] \nonumber \\&-\left\{ { k\nabla T}\right\} \cdot \textbf{n}_{\mathcal {I}_{F\text {S}}}\left[ \!\left[ {{\tau }}\right] \!\right] - \left\{ { k( \nabla \tau )}\right\} \cdot \textbf{n}_{\mathcal {I}_{F\text {S}}}\left[ \!\left[ {{T}}\right] \!\right] \nonumber \\&+ \eta \max (k^{-}, k^{+}) \left[ \!\left[ {{ T}}\right] \!\right] \cdot \left[ \!\left[ {{ \tau }}\right] \!\right] \nonumber \\&- \left\{ { l \textbf{I} }\right\} \textbf{n}_{\mathcal {I}_{F\text {S}}}\cdot \left[ \!\left[ {{\boldsymbol{u}}}\right] \!\right] \quad \textrm{d} S . \end{aligned}$$

It can be obtained simply by a consistent replication of all fluxes and penalty terms from the bulk forms, as defined in form (17), at the interface $\mathcal {I}_{F\text {S}}(t)$.
